# Implementing medical revalidation in the United Kingdom: Findings about organisational changes and impacts from a survey of Responsible Officers

**DOI:** 10.1177/0141076816683556

**Published:** 2017-01-13

**Authors:** Kieran Walshe, Alan Boyd, Marie Bryce, Kayleigh Luscombe, Abigail Tazzyman, John Tredinnick-Rowe, Julian Archer

**Affiliations:** 1 Alliance Manchester Business School, University of Manchester, Manchester M15 6PB, UK; 2 Collaboration for the Advancement of Medical Education Research & Assessment (CAMERA), Plymouth University Peninsula Schools of Medicine & Dentistry, Plymouth PL4 8AA, UK

**Keywords:** Professional regulation, medical revalidation, quality of care, appraisal, evaluation

## Abstract

**Objective:**

To describe the implementation of medical revalidation in healthcare organisations in the United Kingdom and to examine reported changes and impacts on the quality of care.

**Design:**

A cross-sectional online survey gathering both quantitative and qualitative data about structures and processes for medical revalidation and wider quality management in the organisations which employ or contract with doctors (termed ‘designated bodies’) from the senior doctor in each organisation with statutory responsibility for medical revalidation (termed the ‘Responsible Officer’).

**Setting:**

United Kingdom

**Participants:**

Responsible Officers in designated bodies in the United Kingdom. Five hundred and ninety-five survey invitations were sent and 374 completed surveys were returned (63%).

**Main outcome measures:**

The role of Responsible Officers, the development of organisational mechanisms for quality assurance or improvement, decision-making on revalidation recommendations, impact of revalidation and mechanisms for quality assurance or improvement on clinical practice and suggested improvements to revalidation arrangements.

**Results:**

Responsible Officers report that revalidation has had some impacts on the way medical performance is assured and improved, particularly strengthening appraisal and oversight of quality within organisations and having some impact on clinical practice. They suggest changes to make revalidation less ‘one size fits all’ and more responsive to individual, organisational and professional contexts.

**Conclusions:**

Revalidation appears primarily to have improved systems for quality improvement and the management of poor performance to date. There is more to be done to ensure it produces wider benefits, particularly in relation to doctors who already perform well.

## Background

The introduction of medical revalidation in 2012,^1^ after over a decade of protracted policy development and debate,^2^ has been described as the most important change to the way that medical professionals are regulated in the United Kingdom for 100 years.^3^ Put simply, it requires all licenced doctors ‘to demonstrate on a regular basis that they are up to date and fit to practise in their chosen field and able to provide a good level of care’.^4^

Medical revalidation was intended to address long-standing concerns about the accountability of doctors and the quality of medical care,^5,6^ which became the subject of close public and political scrutiny following a number of inquiries into major failures.^7^ The purpose of revalidation has not always been clear^8^ but two main aims have featured – that it should deal more effectively with poor or unacceptable practice, and that it should help doctors to improve their practice. There has been extensive professional scepticism^9^ about whether it can achieve these aims, and concern about its costs which were estimated by the Department of Health as £97 million a year.^10^

For both doctors and organisations, medical revalidation involves important changes to the way that medical performance is managed and assured. It requires healthcare organisations which employ doctors to introduce some new processes for dealing with issues of medical quality and performance and to consider how these relate to existing processes. Revalidation requires every licensed doctor to be connected to one designated body, which is usually their main employer. They must collect, report and reflect on information about various aspects of their performance through an annual appraisal. Healthcare organisations must, through a nominated Responsible Officer, make informed decisions every five years about whether to recommend to the General Medical Council that a doctor be revalidated, and thus allowed to continue to practise. This presumes the existence of a number of organisational systems for managing medical performance, such as clinical audit/quality improvement, continuing professional development, incident reporting and investigation, and complaints management. One of the potential impacts of the introduction of revalidation is that it would lead to changes in these systems for managing medical performance and the way they are used.

Both the Department of Health’ Policy Research Programme and the General Medical Council^11^ have commissioned research studies of the implementation of medical revalidation. The General Medical Council has recently commissioned Sir Keith Pearson, Chair of the General Medical Council's Revalidation Advisory Board, to lead a review of revalidation.^12^

## Methods

We conducted a survey of all Responsible Officers in the UK between June and September 2015. We collected information about how revalidation has been implemented and what resources have been required, how it has interacted with or influenced appraisal and other organisational systems for managing medical performance, what impact it is perceived to have had on these systems and on clinical practice, and how revalidation recommendations have been made. We also sought the views of Responsible Officers about the implementation of revalidation and their suggestions for improving revalidation in the future.

A significant proportion of Responsible Officers fulfil the role for more than one designated body, so to avoid survey overload, we surveyed each of these Responsible Officers only once about the designated body with the greatest number of doctors. A total of 595 survey invitations were issued to Responsible Officers across the UK, and 374 completed survey responses were received (response rate 63%). The response rate did not differ significantly between the countries of the UK or the different regions within England, or between designated bodies who self-reported higher or lower annual appraisal rates in their returns to NHS England, suggesting the responses were broadly representative of designated bodies generally. There was however a significantly lower response rate (just under 50%) from Responsible Officers responsible for designated bodies with less than 20 doctors and from Responsible Officers for locum agencies.

We analysed numeric and Likert scale data from survey respondents using frequency tables and cross-tabulations, identifying statistically significant differences through Chi-square tests. We conducted thematic analyses of free text comments. Themes were identified inductively in relation to each question and then compared across questions in order to identify common, cross-cutting themes.

## Results

### The role of Responsible Officers

Responsible Officers have statutory responsibility for implementing medical revalidation. We found that a high proportion of Responsible Officers are in very senior, board level roles in their designated bodies (64% were the medical director, 6% were an associate or deputy medical director, 8% were consultants and 17% had other roles such as chief executive, deputy chief executive, postgraduate dean, etc.). On average, Responsible Officers had been working in or with their designated body for 10 years. About 15% of Responsible Officers were responsible for multiple designated bodies, with almost 5% being responsible for three or more designated bodies. They reported spending an average of 5.1 hours a week on revalidation.

**Figure 1.**
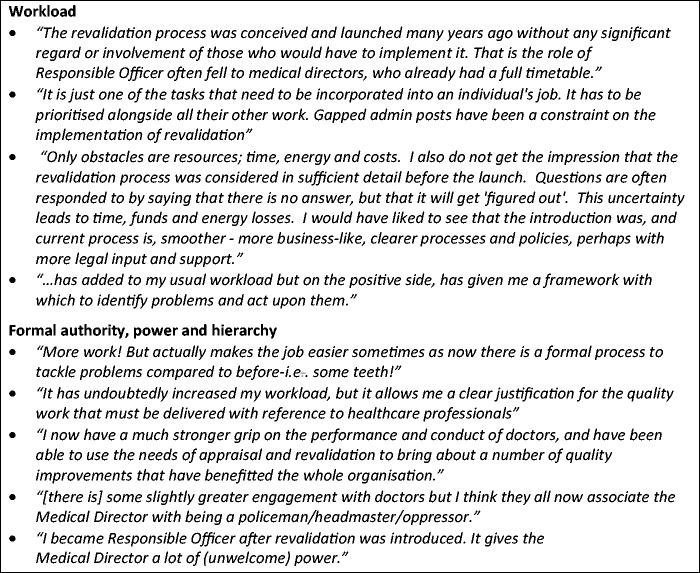
The role of Responsible Officers.

Figure 1 provides some illustrative comments from Responsible Officers about their role. First, they emphasise the workload involved for them personally and for colleagues, and the administrative resource commitment required from their organisations (which was not always forthcoming). Second, they comment on the increased authority and hierarchical power which the Responsible Officer role has given them. Some see this positively – as allowing them to address concerns about quality more effectively, and giving them some leverage for change – but others express concern that it changes the professional, collegial relationship they have had with other doctors and that the powers could be misused.

### Organisational mechanisms for quality assurance or improvement

**Figure 2.**
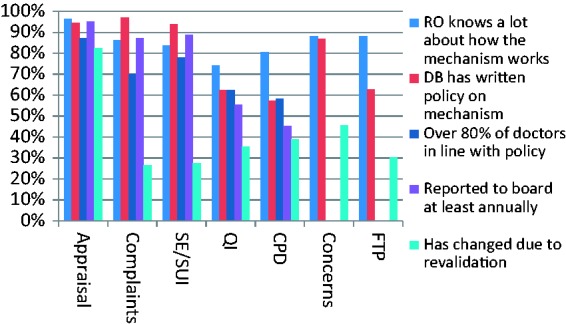
Organisational mechanisms for quality assurance or improvement, and the impact of revalidation.

We asked Responsible Officers a common set of questions about five mechanisms for quality assurance or improvement which are used in the appraisal or revalidation process: appraisal, complaints, significant events or serious untoward incidents, quality improvement/clinical audit and continuing professional development. We also asked about two mechanisms for dealing with poor performance: arrangements for doctors who give cause for concern and formal fitness to practise referral. We were interested in how institutionalised or embedded these mechanisms were, so we asked about the Responsible Officer's knowledge of each one, about whether there was a written policy about it, about compliance by doctors with that policy and about whether the designated body's board received reports about the mechanism. We also asked if each mechanism had changed due to revalidation. The results are shown in Figure 2.

It can be seen that three of these mechanisms – appraisal, complaints and serious untoward incidents – are reported to be well institutionalised with high levels of knowledge about them reported by Responsible Officers, organisations having written policies, high compliance reported with those policies and information about these mechanisms being reported to the organisation’s board. However, two – quality improvement/clinical audit and continuing professional development – have generally lower levels of reported institutional embedding. It can also be seen that Responsible Officers also report high levels of knowledge of arrangements for managing concerns and dealing with fitness to practise.

**Figure 3.**
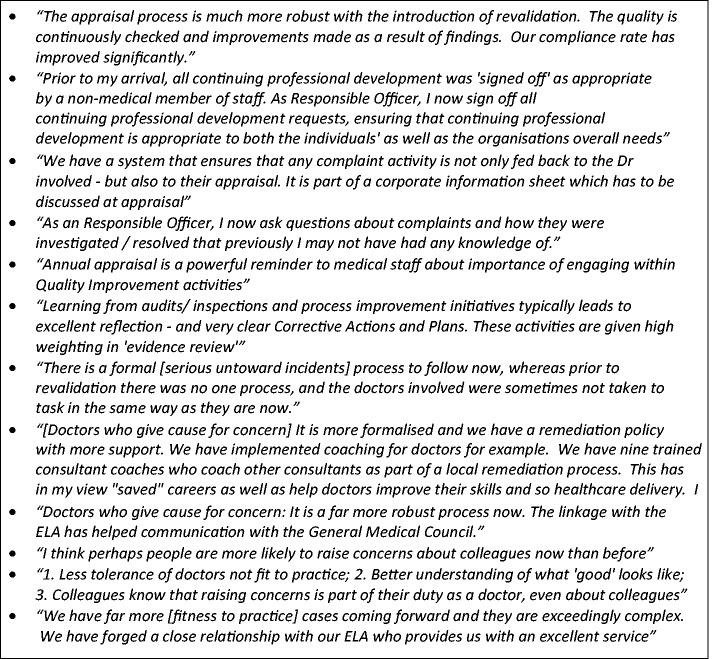
Reported changes in organisational mechanisms for quality assurance or improvement due to the introduction of medical revalidation.

As the graph shows, 82% of Responsible Officers reported that the way appraisals were done had changed due to the introduction of medical revalidation, and 46% similarly reported changes in the arrangements for dealing with doctors causing concern. Between 25% and 40% of Responsible Officers reported changes in the other mechanisms shown in Figure 2. Some examples of their descriptions of these changes are in Figure 3. Across all these systems for quality assurance and improvement, Responsible Officers tended to report that there was greater engagement or participation, that systems were more robust and more formalised, that records of these activities were better kept, that action planning and follow-up in these systems was better and that there was more sharing of information across these systems and within the organisation. Overall, the introduction of revalidation had had some wider effects on the way that organisations deal with quality assurance and improvement, particularly in relation to doctors’ performance.

About 34% of Responsible Officers reported that there had been more cases of doctors causing concern coming to light since the introduction of revalidation, and 26% of Responsible Officers said there were more fitness to practise case referrals to the General Medical Council. Almost all of the rest said there had been no change in the rates of these cases – only 3% said rates of doctors causing concern or fitness to practise referral had fallen.

### Decision-making on revalidation recommendations

The formal revalidation recommendation from the Responsible Officer to the General Medical Council can be to revalidate, to defer revalidation on grounds of insufficient information or an ongoing local process (such as an investigation) or to report non-engagement on the part of the doctor. There is no option to recommend deferring or withholding revalidation because of the doctor’s performance.^13^

We asked Responsible Officers about how they arrived at revalidation recommendations and what recommendations they had made. They reported that information from all the mechanisms for quality assurance or improvement discussed earlier was usually used in these decisions. We found that 88% of Responsible Officers typically reviewed documentation as part of their decision process; with 41% of Responsible Officers reporting that they usually discussed cases with a colleague and 22% reporting that cases were formally reviewed by a committee (Responsible Officers could say they used more than one of these methods, and it was common for Responsible Officers to report that more difficult or complex cases were discussed formally or informally). The Responsible Officer is personally responsible in statute for the revalidation recommendation but 26% of responding Responsible Officers said they, on some occasions, confirmed a recommendation made by someone else, who had typically reviewed the case documentation, etc.

The threshold for making a positive recommendation to revalidate seems ambiguous – 28% of Responsible Officers reported that they had made a positive revalidation recommendation while still having residual concerns about a doctor. Responsible Officers can recommend deferral either because they have insufficient information in the portfolio of supporting information (78% reported they had done so about at least one doctor) or because there is some form of ongoing local process such as an investigation (44% had done so again about at least one doctor). The only other option for Responsible Officers is to make a recommendation of non-engagement which can lead to the General Medical Council taking away the doctor’s licence to practise and 27% reported making at least one such recommendation.

**Figure 4.**
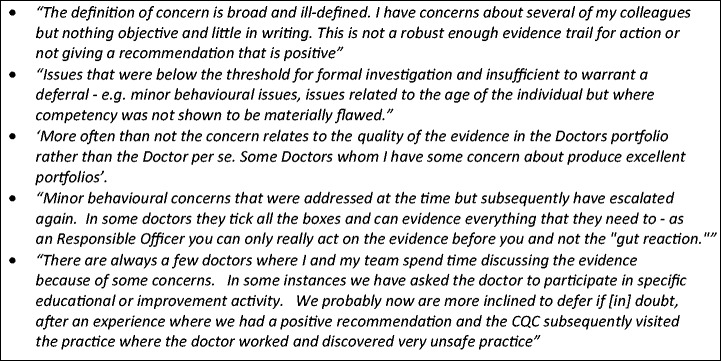
Challenges in arriving at revalidation recommendations.

Responsible Officers commented on the challenges and uncertainties of making revalidation recommendations (Figure 4). First, there was uncertainty about the quality and accuracy of the supporting information in the portfolio from the doctor, and some Responsible Officers expressed reservations about its completeness. Second, there was uncertainty about how or whether to use ‘soft intelligence’ about the doctor if they had it, especially when the portfolio of supporting information seemed adequate but their personal knowledge of the doctor gave them some cause for concern. Third, there was uncertainty about what recommendation to make in the cases of doctors where there were concerns about their performance but which had not yet triggered any formal process. It seemed that deferral was being used in such cases, though this is not in accordance with the General Medical Council guidance.

### Impact on clinical practice and quality of care

**Figure 5.**
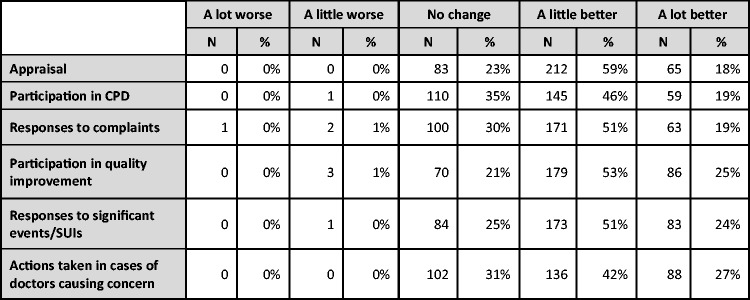
Impact of mechanisms for quality assurance or improvement on clinical practice.

We asked Responsible Officers for their views on what impact the various mechanisms for quality assurance and improvement listed earlier (in Figure 2) had had on clinical practice, and their responses are shown in Figure 5. All mechanisms were seen as having little or no negative impact, and while between one-quarter and one-third of Responsible Officers reported that these mechanisms had no impact, between two-thirds and three-quarters reported positive impacts on clinical practice.

Some Responsible Officers gave examples of such changes, of which most (94%) were positive and few (6%) were negative. Positive reported changes related mostly to improved clinical standards, reflective practice, quality assurance and follow-up on instances of poor performance or behaviour. Negative reported changes related to these activities reducing the time doctors had to spend on direct patient care, and them contributing to or causing a less open, supportive or nurturing culture in which doctors might be less willing to raise or share problems.

### Improving revalidation

**Figure 6.**
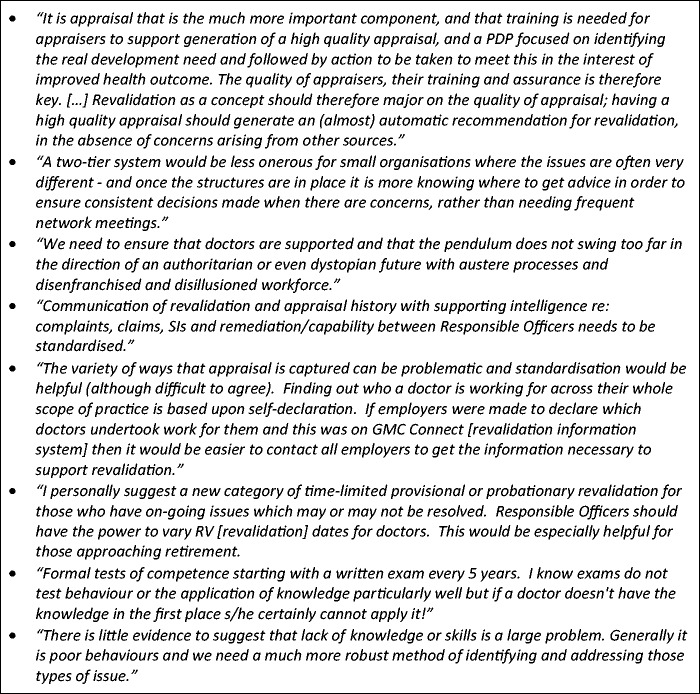
Responsible Officer’s proposals for improving medical revalidation.

Responsible Officers commented extensively about how revalidation could be improved (Figure 6). First and perhaps most commonly, they saw a need to improve the quality of the annual appraisal process which is central to medical revalidation, through better training for appraisers and more quality assurance. Second, some Responsible Officers believed that there should be greater flexibility to tailor the revalidation model to fit particular organisational or professional contexts. For example, Responsible Officers in small and non-NHS organisations saw revalidation as onerous, bureaucratic and designed for large NHS organisations. They wanted a lighter touch approach. Third, some Responsible Officers identified particular difficulties in completing appraisal and revalidation for doctors who moved frequently between organisations (such as locums) or who had portfolio careers across a number organisations. The Responsible Officer in their designated body was expected to take account of their performance across the whole of their professional practice, but mechanisms for sharing information between organisations or transferring it between Responsible Officers were not seen as being well developed.

## Discussion

Overall, we find that medical revalidation has so far had some important impacts on the way that medical performance is managed and assured – in particular strengthening oversight within organisations (especially those which did not have robust systems in place before) and improving liaison with and communication with the General Medical Council. Of course, our survey is based on self-reports from Responsible Officers and we cannot verify their responses. While our response rate is good, there are some measured differences between respondents and non-respondents which could affect our results, and unmeasured differences could also exist.

We find that revalidation has helped to integrate often diverse sources of information within organisations and has given the Responsible Officer the authority and scope to bring together information on performance and to act upon it. The greatest reported impact has been on appraisal, and many Responsible Officer suggestions for improvement were intended to make appraisal and revalidation work better for doctors who are already performing well. A sizeable minority of Responsible Officers felt that more cases of doctors causing concern had come to light since the implementation of revalidation, and also that there had been more fitness to practise referrals. This is consistent with systems being more robust, but might also be due to Responsible Officers becoming more risk averse, with lower thresholds for raising concerns or taking action.

The introduction of medical revalidation has brought into being the role of the Responsible Officer at organisational level, filled by a senior doctor, with concomitant responsibilities, powers and opportunities. In some designated bodies this seems to be changing relationships between the Responsible Officer and medical colleagues, giving the Responsible Officer greater authority over medical practice. It has also created or strengthened external relationships, between Responsible Officers (in part through the Responsible Officer network meetings they attend) and between Responsible Officers and the General Medical Council.

However, there are concerns about the consistency of medical revalidation within and between organisations and about its coverage, particularly of doctors who do not work predominately in one organisation, but who move between organisations and have portfolio careers. The transfer of information about medical performance between organisations seems problematic. The revalidation model seems to suit large organisations with the capacity to put systems in place but works less well for smaller organisations who employ only a few doctors. The extent to which the Responsible Officer can exercise effective oversight reduces as the ‘organisational distance’ to the doctor or ‘organisational transience’ increases. A key challenge would appear to be balancing economies of scale in revalidation systems and administration against the Responsible Officer having a good knowledge of the doctors and organisations they cover and effective means to influence policy and practice in those organisations.

Moving from a ‘one size fits all’ single model of revalidation to allow some legitimate and appropriate variation in the way the policy is applied seems to have widespread support. This might mean differences in the way revalidation works with organisations having either many or few employed doctors; with organisations where there is a particularly close or particularly distant relationship with employed doctors; with doctors in different fields or specialties, due to the clinical content and nature of their work; and perhaps more controversially with individual doctors according to their past and current performance track record. Many would like to see revalidation made less bureaucratic and time consuming.

## Conclusions

It is very difficult to answer the question of what impact medical revalidation has had or will have on clinical practice and the quality of medical care, and more research on this is needed. There are some early positive indications from this survey, and we are exploring both the costs and benefits of medical revalidation further both qualitative and quantitatively in our ongoing research programme. It seems likely that the impact so far is mostly focused on identifying and dealing with poor performance, and there is more to be done to ensure that revalidation has benefits and impact for doctors who perform well already.

## Declarations

### Guarantor

KW.

### Provenance

Not commissioned; peer-reviewed by Jacky Hayden.
